# Classifying early and late mild cognitive impairment stages of Alzheimer’s disease by fusing default mode networks extracted with multiple seeds

**DOI:** 10.1186/s12859-018-2528-0

**Published:** 2018-12-31

**Authors:** Shengbing Pei, Jihong Guan, Shuigeng Zhou

**Affiliations:** 1 Department of Computer Science and Technology, Tongji University, 4800 Cao An Road, Shanghai, 201800 China; 2 Shanghai Key Lab of Intelligent Information Processing, and School of Computer Science, Fudan University, 220 Handan Road, Shanghai, 200433 China

**Keywords:** Default mode network, Seeding-based analysis, Joint independent component analysis, Classification, Alzheimer’s disease

## Abstract

**Background:**

The default mode network (DMN) in resting state has been increasingly used in disease diagnosis since it was found in 2001. Prior work has mainly focused on extracting a single DMN with various techniques. However, by using seeding-based analysis with more than one desirable seed, we can obtain multiple DMNs, which are likely to have complementary information, and thus are more promising for disease diagnosis. In the study, we used 18 early mild cognitive impairment (EMCI) participants and 18 late mild cognitive impairment (LMCI) participants of Alzheimer’s disease (AD). First, we used seeding-based analysis with four seeds to extract four DMNs for each subject. Then, we conducted fusion analysis for all different combinations of the four DMNs. Finally, we carried out nonlinear support vector machine classification based on the mixing coefficients from the fusion analysis.

**Results:**

We found that (1) the four DMNs corresponding to the four different seeds indeed capture different functional regions of each subject; (2) Maps of the four DMNs in the most different joint source from fusion analysis are centered at the regions of the corresponding seeds; (3) Classification results reveal the effectiveness of using multiple seeds to extract DMNs. When using a single seed, the regions of posterior cingulate cortex (PCC) extractions of EMCI and LMCI show the largest difference. For multiple-seed cases, the regions of PCC extraction and right lateral parietal cortex (RLP) extraction provide complementary information for each other in fusion, which improves the classification accuracy. Furthermore, the regions of left lateral parietal cortex (LLP) extraction and RLP extraction also have complementary effect in fusion. In summary, AD diagnosis can be improved by exploiting complementary information of DMNs extracted with multiple seeds.

**Conclusions:**

In this study, we applied fusion analysis to the DMNs extracted by using different seeds for exploiting the complementary information hidden among the separately extracted DMNs, and the results supported our expectation that using the complementary information can improve classification accuracy.

## Background

Functional Magnetic Resonance Imaging (fMRI) [1, 2] provides a novel perspective for the study of brain functions, which is noninvasive and has high resolution in both space and time. Different from task-based fMRI [3, 4] that studies the brain reacting to stimulus, resting state fMRI [5, 6] studies the inner functional connectivity of brain, which can obtain the change of spontaneous functions in our brain. In resting state, there are several functions in operation and the regions of each function constitute a functional network, i.e., resting state network (RSN).

The approaches to extract RSNs from resting state fMRI data mainly fall in two types: data-based [7, 8] and model-based [9, 10]. Both of the two types of approaches have their own merits and demerits. Data-based methods are data driven. For example, independent component analysis (ICA) [11, 12] assumes the independence of the brain patterns; Sparse representation analysis (SRA) [13, 14] assumes the spatial sparsity of brain patterns, but their performance is also limited by the fully data driven process, because sometimes a brain pattern can be further decomposed into more than one subpattern, which causes the difficulty of recognizing RSNs. While model-based methods manually select a representative signal as reference. For example, seeding-based analysis [15–17] assumes some representative regions as seeds and detects temporal correlation between the selected seeds and the other regions, its performance depends on the selected seeds, but the results are unique. In this paper, we consider seeding-based analysis.

Prior works of resting state fMRI mainly use a single extraction of RSN to explore biomarkers or do classification. However, in task-based fMRI, several works conduct multi-task analysis to improve performance. Calhoun et al. [18] described a two-task fusion of auditory oddball and Sternberg working memory for schizophrenia, which reveals two additional findings, compared to the traditional separate analysis. Remezani et al. [19] reported the fusion of three levels of auditory tasks, and showed that the information across multiple tasks can be usefully combined, Remezani et al. [20] compared SRA and ICA for multi-task analysis, and showed the effectiveness of multi-task analysis, but the fusion techniques need to be further improved. The effectiveness of multi-task analysis lies in that each subtraction related to a task can provide complementary information for the others, even though multi-task fMRI data are acquired from the same subject but not necessary at the same time. Considering that resting state fMRI data can provide RSNs that exist at the same time and on the same subject, so it is more likely to get improved performance by combining multiple RSNs.

In our study, we found that there are four seeds can be used to extract the default mode network [21–24] (the main RSN in resting state) by seeding-based analysis, they are medial prefrontal cortex (MPFC), PCC, LLP and RLP [25]. One idea is to average the signals of the four seeds, and then take the average signal as reference for extraction. In this paper, we try to extract four DMNs with the four seeds separately, which can keep their specificities, and then conduct fusion analysis to combine them. We think that this process can help us look insight the relationship of signals of the four seeds. We test this idea on a data set consisting of early mild cognitive impairment participants (EMCI) and late mild cognitive impairment participants (LMCI) of Alzheimer’s disease [26–28]. Alzheimer’s disease is a neurological, progressive disease, which has a strong impact on the lives of some old people, and gains more and more attention in recent years. As the transition from EMCI to LMCI is irreversible, and means a significant change in the state of a patient, we address the classification of these two stages in this paper.

Our study consists of three major steps. First, seeding-based analysis is used to extract DMNs for EMCIs and LMCIs with four seeds, respectively. Second, joint ICA [29–31] is adopted to fuse all the nonempty combinations of the four DMNs. Third, the mixing coefficients from joint ICA are taken as feature for classification, which is based on nonlinear support vector machine (SVM) [32, 33]. Our findings are as follows: 1) the four DMNs extracted by different seeds for subjects are different, and maps corresponding to different seeds in the most different joint source by fusion capture different functional regions. All these lay the foundation of conducting fusion analysis to integrate DMNs extracted by different seeds. 2) The regions of posterior cingulate cortex (PCC) extractions for EMCI and LMCI show the largest difference. 3) The regions of PCC extraction and right lateral parietal cortex (RLP) extraction can provide complementary information for each other in fusion. Besides, the regions of right lateral parietal cortex (LLP) extraction and RLP extraction also have complementary information for each other. 4) We can improve AD diagnosis by exploiting complementary information of DMNs extracted with multiple seeds.

## Methods

**Fig. 1:**
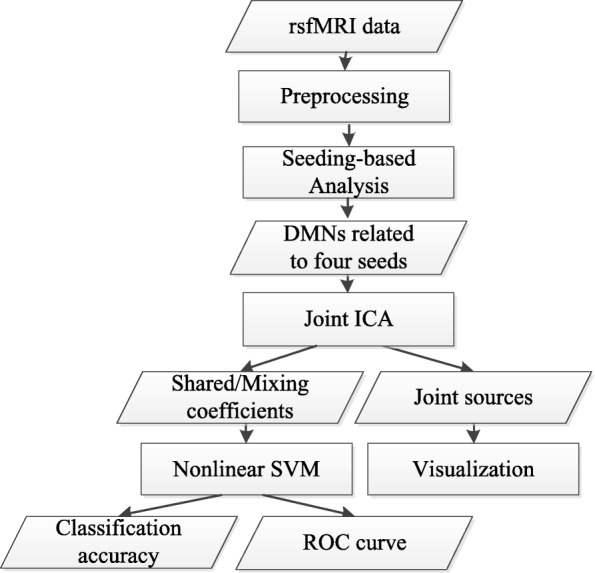
The flowchart of this work

In resting state fMRI, seeding-based analysis can be used to extract various resting state networks from fMRI data. For a RSN, there are often more than one seed available for selection. Concretely, four seeds (MPFC, PCC, LLP, RLP) can be used to extract DMN. It is reasonable to expect that these different extractions contain both complementary and shared information. Our goal is to show that by fusing multiple extractions, we integrate the complementary information from different extractions and enhance the shared information. Consequently, the combined information is more effective in disease diagnosis than using single extraction. To this end, we conduct fusion analysis (i.e., joint ICA) for DMNs extracted by four seeds, and perform nonlinear support vector machine classification based on the coupling shared coefficients. With the combined information, it is expected that the classification accuracy can be improved. The flowchart of this paper is showed in Fig. 1.

### Participants and fMRI data preprocessing

Participants falling to two stages of Alzheimer’s disease are used, so they are split to two groups: early mild cognitive impairment participants (EMCI) and late mild cognitive impairment participants (LMCI). The transition from EMCI to LMCI means an irreversible change of AD. In total, the group of EMCI contains 18 subjects (with 11 females and 7 males, age mean = 72.1667 years and standard deviation (s.d.) = 5.0904 years, Mini Mental State Examination (MMSE) score mean and s.d. are 27.3333 and 1.7150 respectively). The LMCI group also has 18 subjects (5 females and 13 males, age mean and s.d. are 72.2778 and 8.3582 years, MMSE mean and s.d. are 26.5000 and 2.4313). All data were obtained from the Alzheimer’s Disease Neuroimaging Initiative (ADNI) database (http://adni.loni.usc.edu/), which connects researchers with data to study the progression of Alzheimer’s disease since 2004. And the fMRI data are relatively new, which have been added and updated since 2009.

The magnetic resonance image data were acquired using a 3.0T Philips Medical Systems. In the acquisition of functional images, subjects should have eyes open. Each acquisition of functional images consists of 48 contiguous slices, and each slice has a grid of 64×64 (TR = 3000 *ms*, TE = 30 *ms*, flip angle =80^*o*^, voxel size =3.313×3.313×3.313*m,m*^3^). For each subject, a high-resolution, T1-weighted, sagittal MPRAGE, 3D structural image was also captured, which consists of 170 contiguous slices, each of which has a grid of 256×256 (TR = 6.78 *ms*, TE = 3.157 *ms*, flip angle =9.0^*o*^, voxel size =1×1×1.2*m,m*^3^).

All subject data were preprocessed using Statistical Parametric Mapping 8 (SPM8) and Functional Connectivity Toolbox (Conn) on Matlab 2015a. First of all, by using SPM8, the acquired DICOM images were converted to NIFTI format with 140 3D functional images and a 3D structural image for each subject. Then, the first 10 functional images for each subject were discarded to equilibrate the T1 effect. Finally, the preprocessing was done using Conn, the pipeline includes functional realignment and unwarp, functional center to (0,0,0) coordinates, functional slice-timing correction, structural center to (0,0,0) coordinates, structural segmentation and normalization, functional normalization, functional outlier detection, and functional smoothing. It should be noted that the selected standard brain is MNI-space template, the slice order in slice timing is interleaved from top to down, and smoothing is done with an 8-mm Gaussian kernel. After preprocessing, the size of 3D functional images is 91×109×91.

### Seeding-based analysis

Seeding-based analysis is a model-based method because a seed is selected as the model assumption of RSN. The core idea is to evaluate the temporal correlation between the seed and all other regions in brain, and then constitute the corresponding functional connectivity network. In this paper, we perform seeding-based analysis with 4 seeds, MPFC, PCC, LLP and RLP to extract DMNs for EMCI and LMCI participants by using the Conn software, available online at http://www.nitrc.org/projects/conn/. After preprocessing, we do seed-to-voxel analysis, which applies a weighted general linear model to the weighted correlation measures of the condition-specific association between the seed BOLD time series and each voxel BOLD time series. As a result, four DMNs corresponding to the four seeds were extracted for each subject, which are regarded as features for fusion analysis.

### Fusion analysis

In fusion analysis, the extracted features (DMNs) of each subject are concatenated together, so a joint feature is created. Then, a matrix decomposition method (i.e., independent component analysis) is used to represent the joint feature as a linear combination of a set of joint independent sources. The maps of different DMNs in a joint source share a common mixing coefficient. If the fused features are complementary, then the common coefficient could be more discriminative. This constitutes the foundation of the fusion analysis in this paper.

**Fig. 2:**
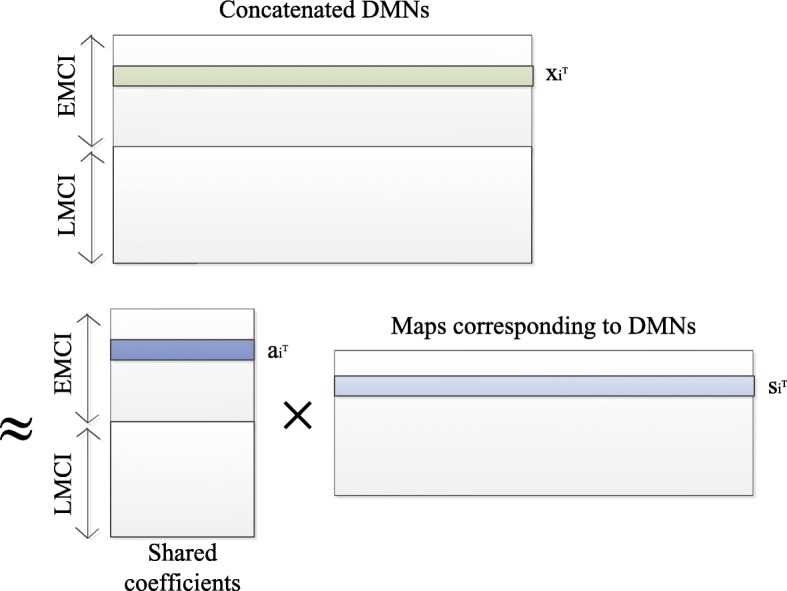
The framework of joint ICA

Figure 2 shows the framework of joint ICA. The model is formulated as *X*=*AS*, where *X*=[*x*_1_,*x*_2_,⋯,*x*_*M*_]^*T*^∈*R*^*M*×*NV*^ is the observation, *x*_*i*_∈*R*^*NV*^ is a joint feature of subject *i*, *M*, *N* and *V* are the number of subjects (including EMCI and LMCI), the number of features (DMNs) and the number of voxels of each subject, respectively. *S*=[*s*_1_,*s*_2_,⋯,*s*_*K*_]^*T*^∈*R*^*K*×*NV*^ is the joint source matrix, *s*_*i*_∈*R*^*NV*^ is the *i*-th joint source, and *K* is the number of joint independent sources. *A*=[*a*_1_,*a*_2_,⋯,*a*_*M*_]^*T*^∈*R*^*M*×*K*^ is the common mixing coefficient matrix, where *a*_*i*_∈*R*^*K*^ is a much shorter vector (compared to the joint feature *x*_*i*_) corresponding to subject *i*, which is taken as a feature for classification. The algorithm to solve this model is generative: First, constructing a statistic to represent the independence of sources, then maximizing or minimizing the statistic to find a matrix *W* that is an approximation of *A*^−1^, *S* is approximated by *WX*. Here, we usee the Infomax algorithm [34, 35] to solve the model, which aims at minimizing the mutual information of the joint independent sources, and is proved to be effective for fMRI data. The optimal approach to estimate the value of *K* is an open issue, although MDL algorithm [36, 37] can be used to estimate it sometimes, it does not always converge. Here, we set *K* =8 as in [38], and repeat the computation with *K* = 6, 10, 12, 14 and 16, and found that the most significant different joint sources have little change with *K* value, which indicates the choice of *K*=8 is desirable. Joint ICA is done by FIT software, available at http://mialab.mrn.org/software/fit/index.html.

To examine whether the fusion of DMNs extracted by four different seeds can integrate complementary information and enhance shared information, we consider all the combinations of the four DMNs, totally 15 settings: 1 of combining 4 DMNs, 4 of combing 3 of the 4 DMNs, 6 of combing 2 of the 4 DMNs, and 4 of using only one of the 4 DMNs.

### Classification with nonlinear support vector machine

As a result of fusion analysis, each subject is transformed to an 8-dimensional feature vector. If the DMNs extracted by different seeds can be effectively fused, the resulting feature vectors can be used to effectively classify EMCI and LMCI. Here, classification is performed by a nonlinear SVM that is useful for a small number of samples. The radial basis function (RBF) is used as the kernel function, and the two parameters, i.e., penalty parameter *C* and radius of the kernel function *g* are determined by grid search with a step size of 0.5. In addition, we use the ROC curve to measure the classification performance. As the output of SVM is not a probability, we train a sigmoid function following the SVM to generate probability output for test set [39]. The nonlinear SVM is implemented by using Statistical Pattern Recognition Toolbox software, available at http://cmp.felk.cvut.cz/cmp/software/stprtool/.

Both the 18 EMCI subjects and the 18 LMCI subjects are randomly split into two groups: 13 subjects for training and 5 subjects for testing. Such splitting is repeatedly done 100 times, and the final performance result is obtained by averaging the results of the 100 testings. Note that the splitting is done on DMN data, while each input of SVM is an 8-dimensional feature vector obtained by fusion analysis. In training, the 8-dimensional feature vectors are obtained by joint ICA. In testing, we set the joint sources from joint ICA as basic sources, and the 8-dimensional feature vectors are obtained by mapping the joint DMN data to the bases, which is solution of a least square problem.

All the 15 combinations of the four seeds of DMNs are tested. Performance comparison is done to check the complementary effect of the seeds for Alzheimer’s disease diagnosis.

## Results

### DMNs extracted by seeding-based analysis

**Fig. 3:**
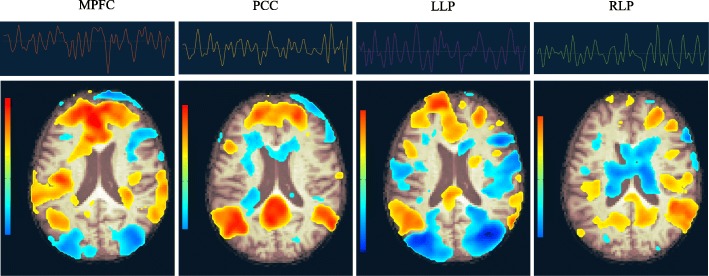
Seeding-based analysis results of DMNs for subject 100_S_4556. From left to right, the sub-figures are the time series and extracted DMNs corresponding to the four seeds MPFC, PCC, LLP and RLP, respectively. The first row are timeseries and the second row are DMNs

Figure 3 shows the source time series and extracted DMNs with the four seeds MPFC, PCC, LLP and RLP for subject 100_S_4556 (randomly selected). The results show that the signals extracted by 4 different seeds are quite variant, and even for the same DMN, different seeds can capture different characteristics of DMN, which lays down the foundation of conducting fusion analysis to combine the complementary information of different DMNs.

### The most significant joint source from joint ICA

**Fig. 4:**
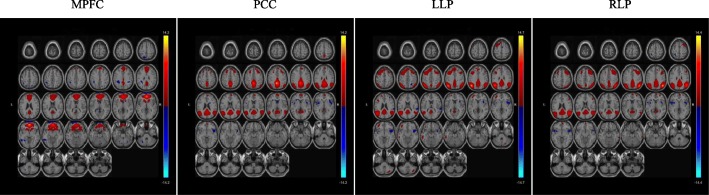
Most different joint source between EMCI and LMCI from the joint ICA of DMNs extracted by MPFC, PCC, LLP and RLP. From left to right, the sub-figures are maps corresponding to MPFC, PCC, LLP and RLP respectively

As an example, Fig. 4 shows the most significantly different joint source for the fusion of all the four DMNs extracted by the four seeds (MPFC, PCC, LLP, RLP) between EMCI and LMCI. From left to right, the maps correspond to the results of MPFC, PCC, LLP and RLP respectively. It can be seen that the maps corresponding to different seeds show obvious difference, and are concentrated on the regions of the corresponding seeds. Particularly, the maps corresponding to LLP and RLP look like each other more than the other maps. In fusion analysis, the four maps share a common coefficient, which is determined by the four maps. If the information from DMNs extracted by different seeds can be fused, the performance of classification by common coefficients can be improved.

### Classification

**Fig. 5:**
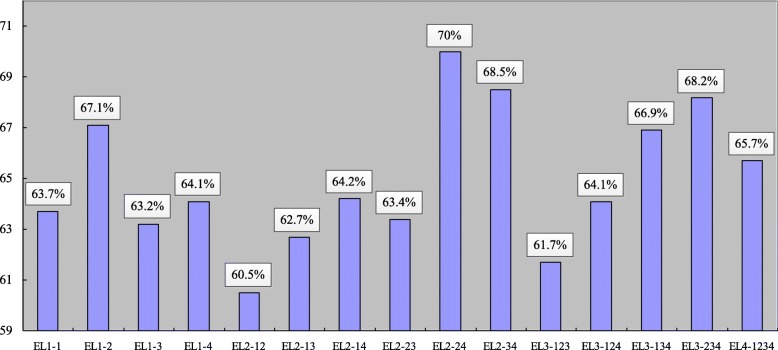
Classification accuracy results of the 15 combinations of the four DMNs extracted seeds MPFC, PCC, LLP and RLP. We label MPFC, PCC, LLP and RLP by ‘1, ‘2’, ‘3’ and ‘4’ respectively. EL1-1, EL1-2, EL1-3, EL1-4 represent the cases using only one of the four seeds; EL2-12, EL2-13, EL2-14, EL2-23, EL2-24, EL2-34 represent the cases using two seeds; EL3-123, EL3-124, EL3-134, EL3-234 represent the cases using three seeds, and EL4-1234 represent the case using all the four seeds

Figure 5 shows classification accuracies of all combinations of the four DMNs extracted by the four seeds. As it can be seen, for one-seed cases, PCC obtains the best performance (67.1%), which indicates the regions of PCC extraction in the DMNs of AD subjects are more discriminative. For the multiple seeds cases, the combination of PCC and RLP obtains the best performance (70%). Besides, the combination of LLP and RLP also gets improved performance (68.5%), while the other combinations cannot get improved performance in comparison with their sub-combinations. This means that the regions of PCC extraction and RLP extraction can provide complementary information for each other, and the regions of LLP extraction and RLP extraction also have complementary information for each other. However, for the other cases, it is harder to combine the maps in fusion.

**Fig. 6:**
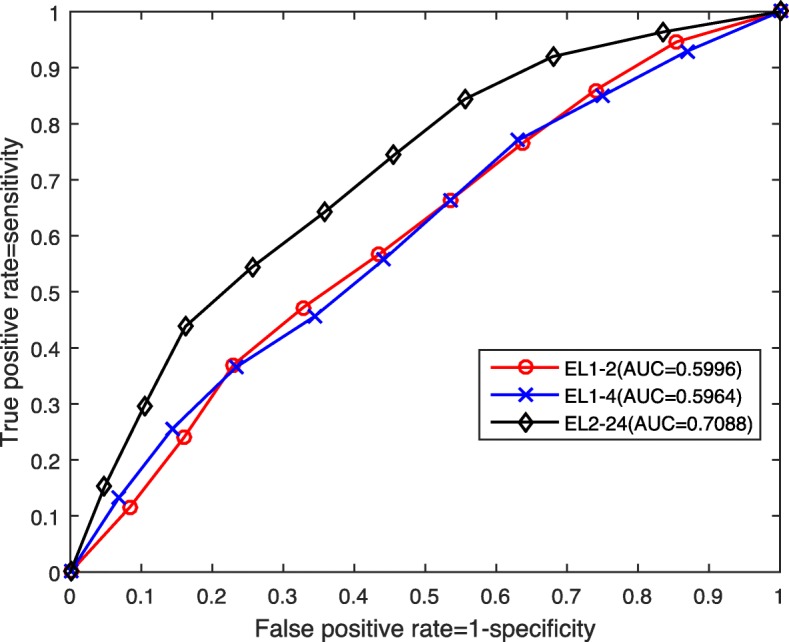
ROC curves and AUC values for PCC, RLP and their combination

**Fig. 7:**
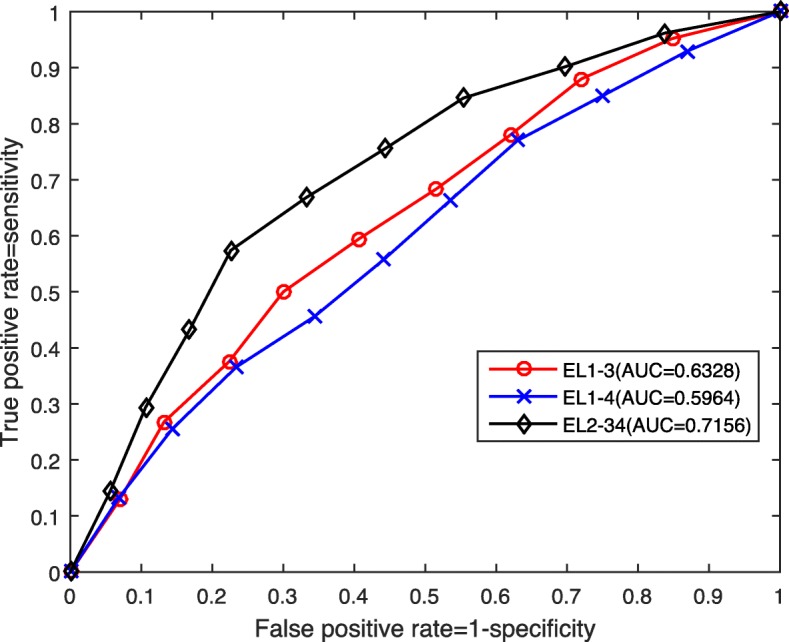
ROC curves and AUC values for the cases of LLP, RLP, and their combination

To further illustrate the classification performance of the cases corresponding to PCC, RLP and their combination, LLP, RLP and their combination, we plot their ROC curves and present their AUC values in Figs. 6 and 7 respectively.

## Discussion and conclusion

In this paper, we first applied seeding-based analysis with four seeds to extract DMNs from resting state fMRI data for two groups of AD subjects (EMCI and LMCI), then performed joint ICA on them, finally trained a nonlinear SVM to classify these two groups of AD subjects with the fused information. The results support our expectation that using complementary information among separately extracted DMNs can improve classification accuracy.

In the classification based on a single seed (without fusion), PCC extraction obtains the best performance. This suggests that if we want to discriminate patients of EMCI and LMCI using one DMN, PCC extraction is the best choice. In our fusion analysis, we found that the maps of LLP and RLP extractions in the joint source show high similarity, and fusing the two extractions can improve classification accuracy by about 5%. This indicates the symmetry and complementarity of the left and right brain functions.

In practice, we often need a unique DMN for each subject to do follow-up research. Now we know PCC and RLP extractions can complement each other, so we can use the RLP extraction to supplement the PCC extraction, which can generate a unique DMN with more complete information.

Our study about the relationship among different seeds is a second-level approach, which is based on the fusion of extracted DMNs instead of straightforwardly analyzing the extracted DMNs. Whether or not the extracted DMNs are complementary is implied in the mixing coefficients, which are short features (rather than thousands of voxels).

For a subject, joint ICA provides an 8-dimensional feature vector that is favorable for classification, and it indeed proves our expectation. However, it is worthy of pointing out that the feature selection is limited by the hypothesis of independence. We believe that with more advanced feature selection methods (e.g. deep neural networks [40, 41]), the accuracy of EMCI and LMCI classification can be further improved.

The experimental results in this paper show that the fusion of DMNs obtained with different seeds is effective. Moreover, the idea of this work can be extended to multiple RSNs, and different RSNs can also be fused to improve disease diagnosis. The key is to exploit the complementary information among the RSNs.

In summary, we used seeding-based analysis, joint ICA and SVM to improve classification accuracy by combining different DMNs extracted by different seeds over two groups of AD conditional subjects, and found that PCC extraction shows the largest between EMCI and LMCI. Meanwhile, PCC and RLP extractions as well as LLP and RLP extractions can complement each other in fusion. Our future work will focus on more advanced feature selection methods to improve classification accuracy under the fusion analysis framework.

## Abbreviations

AD: Alzheimer’s disease
ADNI: Alzheimer’s Disease Neuroimaging Initiative
AUC: Area under curve
BOLD: Blood oxygenation level dependent
DMN: Default mode network
EMCI: Early mild cognitive impairment
fMRI: Functional magnetic resonance imaging
ICA: Independent component analysis
LLP: Left lateral parietal cortex
LMCI: Late mild cognitive impairment
MMSE: Mini mental state examination
MPFC: Medial prefrontal cortex
PCC: Posterior cingulate cortex
RBF: Radial basis function
RLP: Right lateral parietal cortex
ROC: Receiver operating characteristic
RSN: Resting state network
SRA: Sparse representation analysis
SVM: Support vector machine
